# Human microbiota dysbiosis after SARS-CoV-2 infection have the potential to predict disease prognosis

**DOI:** 10.1186/s12879-023-08784-x

**Published:** 2023-11-29

**Authors:** Jie Zhou, Xiping Yang, Yuecong Yang, Yiru Wei, Dongjia Lu, Yulan Xie, Hao Liang, Ping Cui, Li Ye, Jiegang Huang

**Affiliations:** 1 Guangxi Key Laboratory of AIDS Prevention and Treatment & School of Public Health, Guangxi Medical University, Nanning, Guangxi China; 2 Guangxi Universities Key Laboratory of Prevention and Control of Highly Prevalent Disease, Nanning, Guangxi China; 3 Life Science Institute, Guangxi Medical University, Nanning, Guangxi China; 4 Collaborative Innovation Centre of Regenerative Medicine and Medical BioResource Development and Application Co-Constructed By the Province and Ministry, Nanning, Guangxi China

**Keywords:** SARS-CoV-2/COVID-19, Human microbiota, α-diversity, Key microbiota, Machine learning

## Abstract

**Background:**

The studies on SARS-CoV-2 and human microbiota have yielded inconsistent results regarding microbiota α-diversity and key microbiota. To address these issues and explore the predictive ability of human microbiota for the prognosis of SARS-CoV-2 infection, we conducted a reanalysis of existing studies.

**Methods:**

We reviewed the existing studies on SARS-CoV-2 and human microbiota in the Pubmed and Bioproject databases (from inception through October 29, 2021) and extracted the available raw 16S rRNA sequencing data of human microbiota. Firstly, we used meta-analysis and bioinformatics methods to reanalyze the raw data and evaluate the impact of SARS-CoV-2 on human microbial α-diversity. Secondly, machine learning (ML) was employed to assess the ability of microbiota to predict the prognosis of SARS-CoV-2 infection. Finally, we aimed to identify the key microbiota associated with SARS-CoV-2 infection.

**Results:**

A total of 20 studies related to SARS-CoV-2 and human microbiota were included, involving gut (*n* = 9), respiratory (*n* = 11), oral (*n* = 3), and skin (*n* = 1) microbiota. Meta-analysis showed that in gut studies, when limiting factors were studies ruled out the effect of antibiotics, cross-sectional and case–control studies, Chinese studies, American studies, and Illumina MiSeq sequencing studies, SARS-CoV-2 infection was associated with down-regulation of microbiota α-diversity (*P* < 0.05). In respiratory studies, SARS-CoV-2 infection was associated with down-regulation of α-diversity when the limiting factor was V4 sequencing region (*P* < 0.05). Additionally, the α-diversity of skin microbiota was down-regulated at multiple time points following SARS-CoV-2 infection (*P* < 0.05). However, no significant difference in oral microbiota α-diversity was observed after SARS-CoV-2 infection. ML models based on baseline respiratory (oropharynx) microbiota profiles exhibited the ability to predict outcomes (survival and death, Random Forest, AUC = 0.847, Sensitivity = 0.833, Specificity = 0.750) after SARS-CoV-2 infection. The shared differential *Prevotella* and *Streptococcus* in the gut, respiratory tract, and oral cavity was associated with the severity and recovery of SARS-CoV-2 infection.

**Conclusions:**

SARS-CoV-2 infection was related to the down-regulation of α-diversity in the human gut and respiratory microbiota. The respiratory microbiota had the potential to predict the prognosis of individuals infected with SARS-CoV-2. *Prevotella* and *Streptococcus* might be key microbiota in SARS-CoV-2 infection.

**Supplementary Information:**

The online version contains supplementary material available at 10.1186/s12879-023-08784-x.

## Background

SARS-CoV-2 infection can manifest as asymptomatic, mild symptoms, acute respiratory symptoms, and even respiratory failure [1]. Elderly individuals and those with weakened immune function are prone to experiencing severe complications from SARS-CoV-2 [2]. Studies have suggested that auxiliary proteins encoded by SARS-CoV-2, such as ORF9c and ORF10, may play a crucial role in virus replication and immune evasion, contributing to the pathogenic mechanism of SARS-CoV-2 [3]. Additionally, research indicates that respiratory microbiota could influence the progression of COVID-19 by promoting local mucosal inflammation, modifying pathogen-associated molecular patterns, and depleting beneficial bacteria [4–6]. These findings suggest that human microbiota might have an important role in SARS-CoV-2 infection.

So far, there have been many studies exploring the relationships between SARS-CoV-2 infection and human microbiota. However, whether SARS-CoV-2 infection affects human microbiota is still controversial, especially regarding the α-diversity index used to evaluate the integrity of the microbiota. One study showed a significant up-regulation of the α-diversity of the gut microbiota in SARS-CoV-2 infected individuals [7]. Conversely, several other studies suggested a significant down-regulation of the α-diversity of the gut microbiota following SARS-CoV-2 infection [8–12], while some studies found no change in the α-diversity of the gut microbiota after infection [13, 14]. Similar inconsistencies can be observed in studies on SARS-CoV-2 infection and respiratory microbiota [15–17]. The alteration of human microbiota caused by SARS-CoV-2 infection has been utilized for diagnosing the severity of the disease [18], but there are limited research reports on whether it can predict disease prognosis. Studies have shown that human microbiota plays an important role in immune regulation following SARS-CoV-2 infection and can serve as biomarkers of the infection [19]. Different studies have identified different key human microbiota associated with SARS-CoV-2 [9, 20, 21]. Therefore, further investigation is necessary to determine the key human microbiota associated with SARS-CoV-2.

To address the aforementioned issues, we reviewed the existing studies on SARS-CoV-2 and human microbiota in the Pubmed and Bioproject databases and extracted the available raw 16S rRNA sequencing data of human microbiota. Firstly, we utilized meta-analysis and bioinformatics methods to assess the impact of SARS-CoV-2 on the α-diversity of human microbiota. Secondly, ML was employed to evaluate the ability of the microbiota to predict the prognosis of SARS-CoV-2 infection. Finally, we aimed to identify the key microbiota associated with SARS-CoV-2 infection.

## Methods

### Data sources and search strategy

PubMed and BioProject were searched using keywords, medical subject headings (MeSH) terms, and synonyms for SARS-CoV-2 and human microbiota from inception to Oct 29, 2021. Two independent reviewers evaluated each study, and an independent reviewer reviewed all studies. The exclusion criteria for studies were as follows: 1. Non-microbiota studies; 2. Non-SARS-CoV-2 studies; 3. No population studies; 4. Reviews, Commentaries, etc.; 5. Non-16S rRNA gene sequence studies; 6. Raw sequences not open access; 7. The raw data of the studies cannot distinguish between cases and controls; 8. Studies without negative control samples.

### Processing of raw data and calculation of α-diversity

Raw 16S rRNA sequencing sequences download and conversion using the sratoolkit tool developed by NCBI. The Fastp software for quality control of the original sequencing data and then imported the sequences into QIIME 2 (version 2021.11) [22]. Quality filtering of raw sequences using the q2-demux plugin, followed by DADA2 [23] (via q2-dada2) for denoising and generation of amplicon sequence variants (ASV) table. All ASV were aligned with Mafft [24] (q2-alignment) and used to construct a phylogenetic tree with fasttree2 [25] (q2-phylogeny). Species annotation of ASV was performed with silva-138 99% OTU reference sequences pre-trained with classify-sklearn Naive Bayes classifier [26] and q2-feature-classifier plugin [27].

The generated ASV table, phylogenetic tree, and species annotation files were imported into R 4.2.2., and the vegan package was used to normalize the sample sequence according to the minimum sample sequence number so that each sample could be compared under the same sequence number. Microbial α-diversity indexes [Observed, Chao1, ACE, Fisher, Shannon, Simpson, Invsimpson, and Phylogenetic diversity (PD) indexes] were calculated by the R microeco (https://chiliubio.github.io/microeco_tutorial/), https://chiliubio.github.io/microeco_tutorial/https://chiliubio.github.io/microeco_tutorial/ [28] package. According to the above steps, all available studies were processed respectively, and then quantitative consolidation of meta-analysis was performed.

### Meta-analysis

Quantitative synthetic analysis of α-diversity indices from different sources using Review Manager 5.3 software (Copenhagen: The Nordic Cochrane Centre, The Cochrane Collaboration, 2014.). For continuous variables, mean and standard deviation (*SD*) were used as analytical statistics, and each effect size was represented by a 95% *CI* value. The Fixed-effect models were used to quantitatively combine the α-diversity of each study. The heterogeneity of the included studies was tested by the *Q* test and *I*^*2*^ test. If there was significant heterogeneity among studies (*Q* test of *P* < 0.05 or *I*^*2*^ ＞ 50%), subgroup analyses were performed for included studies to assess the sources of heterogeneity. Limiting factors for subgroup analysis included antibiotic, study type (Cross-sectional studies and case–control studies were grouped into one category, and longitudinal studies into another), country (Chinese or American studies), sequencing platform (Illumina MiSeq), and sequencing region (V3-V4 or V4). Sensitivity analyses were performed by removing the studies with the largest sample size. Funnel plots were drawn to describe publication bias.

### Machine learning (ML) strategy

We used the microbiological feature screening tool linear discriminant analysis effect size (LEfSe, linear discriminant analysis [LDA] score > 2, *P* < 0.05) in the microco package to identify the different ASV between groups and then construct the ML models. We used the caret package in R to build and evaluate ML models. We considered eight models related to kernel learning (support vector machine [SVM]), ensemble (random forest [RF] and extreme gradient boosting [XGB]), instance-based (k-nearest neighbor [KNN]), regulation (logistic regression [LR]), Bayesian (naiveBayes [NB]), decision tree (DT), neural networks (NN) algorithms to train the ASV table of the gut microbiota. We randomly divided the ASV tables into training and test datasets in a 7:3 ratio before training. We evaluated the training effects of different ML models by using tenfold cross-validation, and we repeated the process 10 times to obtain optimal parametric modeling. We evaluated the performance of the models in the test dataset from three perspectives: area under the curve [AUC], sensitivity, and specificity.

### Identification of key microbiota

The microeco package in R was employed to identify differential microbiota through the linear discriminant analysis effect size (LEfSe) method (LDA score ＞ 2, *P* < 0.05). Cytoscape 3.7.1 was used to plot venn networks to identify shared differential microbiota across studies or different body parts.

### Statistical analysis

For data that meet normality, we analyzed them using the Student's t test. For data that did not meet normality, we analyzed them using the Mann–Whitney U test. All tests were two-sided, and *P* < 0.05 was considered statistically significant.

## Results

### Meta-analysis of human microbiota α-diversity in SARS-CoV-2 infected individuals

**Fig. 1:**
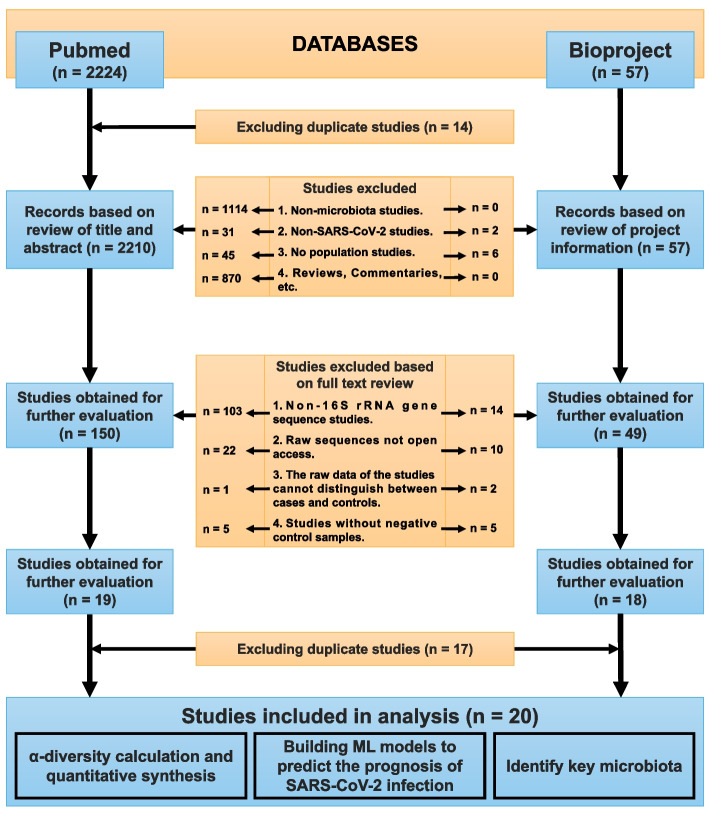
Data mining pipeline

A total of 2224 records from PubMed and 57 records from BioProject were retrieved (Fig. 1). Through a review of existing studies, we found inconsistent results regarding changes in human microbiota α-diversity following SARS-CoV-2 infection across the studies (Supplementary Table 1).

**Fig. 2:**
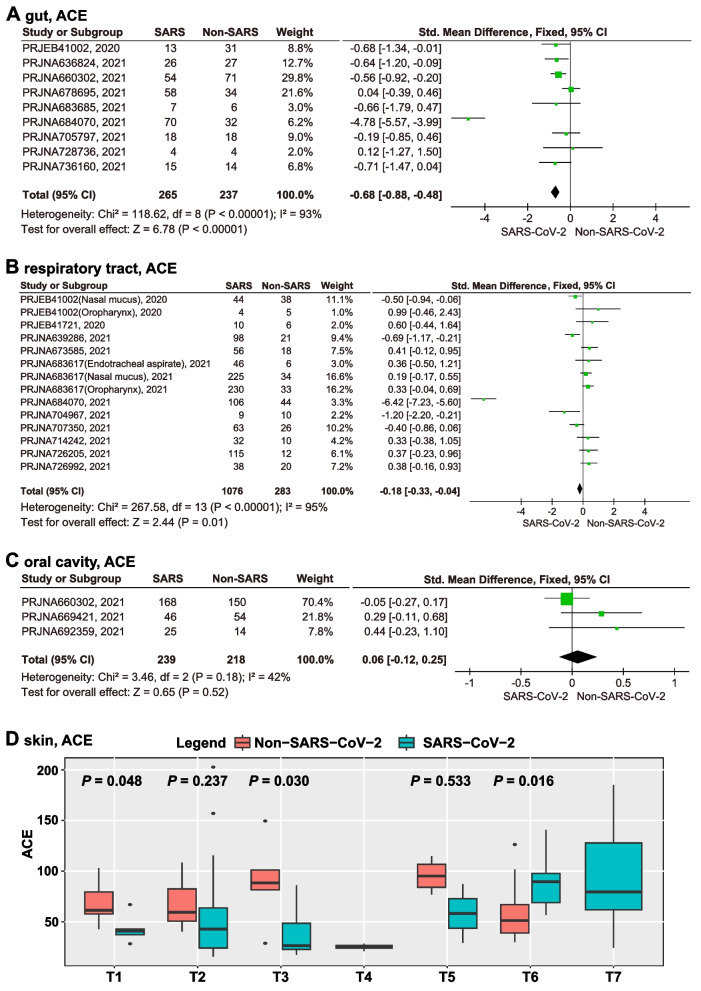
α-diversity analysis (ACE index) of human microbiota between SARS-CoV-2 and non-SARS-CoV-2 infected individuals. The meta-analysis based on the fixed-effects models quantitatively merged studies on gut (**A**), respiratory (**B**), and oral (**C**) studies, respectively. Changes in α-diversity of skin microbiota from observation time point 1 to time point 7 (T1-T7) in SARS-CoV-2 and non-SARS-CoV-2 infected individuals (**D**)

Twenty studies, including raw sequences of the gut, respiratory, oral, and skin microbiota, were included in the meta-analysis of α-diversity (Supplementary Table 1). Among them, nine studies were related to the gut microbiota, including 265 samples from SARS-CoV-2 infected individuals and 237 samples from non-SARS-CoV-2 infected individuals. Our results showed that the α-diversity of gut microbiota was significantly down-regulated in SARS-CoV-2 infected individuals compared with non-SARS-CoV-2 individuals (Fixed-effect models; ACE, Chao1, Observed, InvSimpson, Fisher, Shannon, Simpson, and PD indexes, *P* < 0.05), but there were significant heterogeneity among studies (Fig. 2A). Therefore, we used subgroup analysis to analyze the sources of heterogeneity. When the limiting factors were studies ruled out the effect of antibiotics (Simpson index, *P* < 0.001, Supplementary Fig. 1A), cross-sectional and case–control studies of published articles (ACE and Fisher indexes, *P* < 0.001; Supplementary Fig. 1B), Chinese studies (Simpson index, *P* < 0.001, Supplementary Fig. 1C), American studies (InvSimpson index, *P* < 0.01, Supplementary Fig. 1D), and Illumina MiSeq studies (ACE, Chao1, Oberved, Fisher, and InvSimpson indexes, *P* < 0.001, Supplementary Fig. 1E), and there were no significant heterogeneity among included studies. These results remained consistent when the studies with the largest sample size were removed.

There were eleven studies related to the respiratory microbiota, including 1,076 samples from SARS-CoV-2 infected individuals, and 283 samples from non-SARS-CoV-2 infected individuals. Analysis based on fixed-effects models showed that the α-diversity of respiratory microbiota was significantly down-regulated in SARS-CoV-2 infected individuals compared with non-SARS-CoV-2 infected individuals (ACE, Chao1, PD, and Simpson indexes, *P* < 0.01; significant heterogeneity; Fig. 2B). Subgroup analyses that included 5 identified cross-sectional and case–control studies still showed that SARS-CoV-2 infection was associated with significant down-regulation of respiratory microbiota α-diversity (PD index, *P* = 0.03, no significant heterogeneity, Supplementary Fig. 2A). However, SARS-CoV-2 infection was not associated with α-diversity when the limiting factor was the V4 sequencing region (Simpson index, *P* = 0.47, no significant heterogeneity, Supplementary Fig. 2B). These results remained consistent when the studies with the largest sample size were removed. The results of other limiting factors were still heterogeneous or the results of sensitivity analysis were unstable, or the number of included studies was less than three.

There were three cross-sectional studies related to oral microbiota, including 239 samples from confirmed and convalescent SARS-CoV-2 infected individuals and 218 samples from non-SARS-CoV-2 infected individuals. All three studies ruled out the effect of antibiotics. Fixed-effects models showed that the α-diversity of oral microbiota were not significantly different in SARS-CoV-2 infected individuals compared with non-SARS-CoV-2 infected individuals (ACE and Chao1 indexes, *P* > 0.05; no significant heterogeneity; Fig. 2C). These results remained consistent when the studies with the largest sample size were removed. Due to the small number of studies, the potential confounding factors were not further analyzed.

Moreover, in a longitudinal study of 81 skin samples, the skin microbiota α-diversity of SARS-CoV-2 infected individuals was significantly lower than that of non-SARS-CoV-2 infected individuals at sampling points 1 (*P*=0.048) and 3 (*P*=0.03). The skin microbiota α-diversity of SARS-CoV-2 infected individuals decreased first and then increased (Fig. 2D).

### ML models based on the human respiratory microbiota could predict the prognosis of SARS-CoV-2 infection

Respiratory (nasopharynx and oropharynx) microbiota study PRJNA683617 provides the outcomes, including death and survival, of hospitalized SARS-CoV-2 infected individuals. Based on LEfSe analysis, we identified differential microbial ASV features between dead and surviving SARS-CoV-2 infected individuals at baseline sampling time, and constructed eight ML models based on these features (Table 1). We found that both the nasopharyngeal and oropharyngeal microbiota have predictive potential for the prognosis of SARS-CoV-2 infected individuals. In the ML models constructed by the nasopharyngeal microbiota, SVM showed the best predictive performance, with AUC, sensitivity, and specificity of 0.781, 0.947, and 0.667, respectively. In the ML models constructed by the oropharyngeal microbiota, RF showed the best predictive performance, with AUC, sensitivity, and specificity of 0.847, 0.833, and 0.750, respectively.

**Table 1:** ML models based on the human respiratory microbiota were used to predict the prognosis of SARS-CoV-2 infection

	Models	AUC	Sensitivity	Specificity
Data: the nasopharyngeal microbiota of SARS-CoV-2 infected individuals; Predicting outcomes: survival (*n*=56) and death (*n*=17).	SVM	0.781	0.947	0.667
	RF	0.772	0.947	0.667
	KNN	0.754	0.842	0.667
	LR	0.518	1.000	0.333
	NB	0.632	0.000	1.000
	DT	0.500	1.000	0.000
	NNET	0.509	1.000	0.333
	XGB	0.737	0.842	0.667
Data: the oropharyngeal microbiota of SARS-CoV-2 infected individuals; Predicting outcomes: survival (*n*=56) and death (*n*=16).	SVM	0.688	0.778	0.750
	RF	0.847	0.833	0.750
	KNN	0.75	0.833	0.500
	LR	0.542	0.000	1.000
	NB	0.604	1.000	0.250
	DT	0.5	1.000	0.000
	NNET	0.451	0.667	0.500
	XGB	0.556	0.944	0.500

### *Prevotella* and *Streptococcus* were shared differential bacteria in gut, respiratory tract, and oral cavity after SARS-CoV-2 infection

**Fig. 3:**
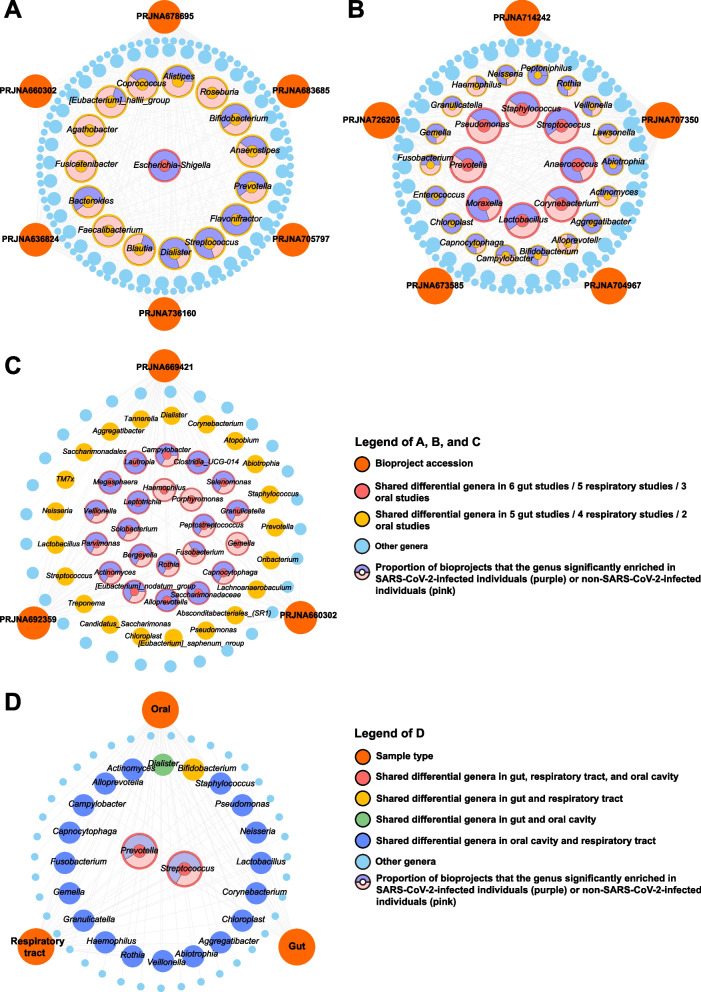
Identification of key genera altered after SARS-CoV-2 infection based on LEfSe and venn networks. **A** Venn network of six cross-sectional and case–control studies of gut genera. **B** Venn network of five cross-sectional and case–control studies of respiratory genera. **C** Venn network of three cross-sectional studies of oral genera. **D** Venn network of gut, respiratory, and oral genera

The above studies suggested that the type of study was one of the sources of heterogeneity. Therefore, we first identified the shared differential bacteria in multiple cross-sectional and case–control studies, and then observed their changing characteristics in longitudinal studies. Through LEfSe and Venn network analysis, we found that the abundance of *Escherichia-Shigella* were significantly different between SARS-CoV-2 and non-SARS-CoV-2 infected individuals in six cross-sectional and case–control gut microbiota studies (Fig. 3A). *Anaerococcus, Corynebacterium, Lactobacillus, Moraxella, Prevotella, Pseudomonas, Staphylococcus,* and *Streptococcus* were shared differential genera in five cross-sectional and case–control respiratory microbiota studies (Fig. 3B). There were 22 shared differential genera such as *Alloprevotella*, *Actinomyces*, and *Bergeyella* in three cross-sectional oral microbiota studies (Fig. 3C). Further, genera with significant differences in more than half of the studies in each sample type were included in the Venn network. It could be seen that the *Prevotella* and *Streptococcus,* which were shared in the gut, respiratory tract, and oral cavity, were enriched in SARS-CoV-2 infected individuals in more than half of the studies (Fig. 3D).

**Fig. 4:**
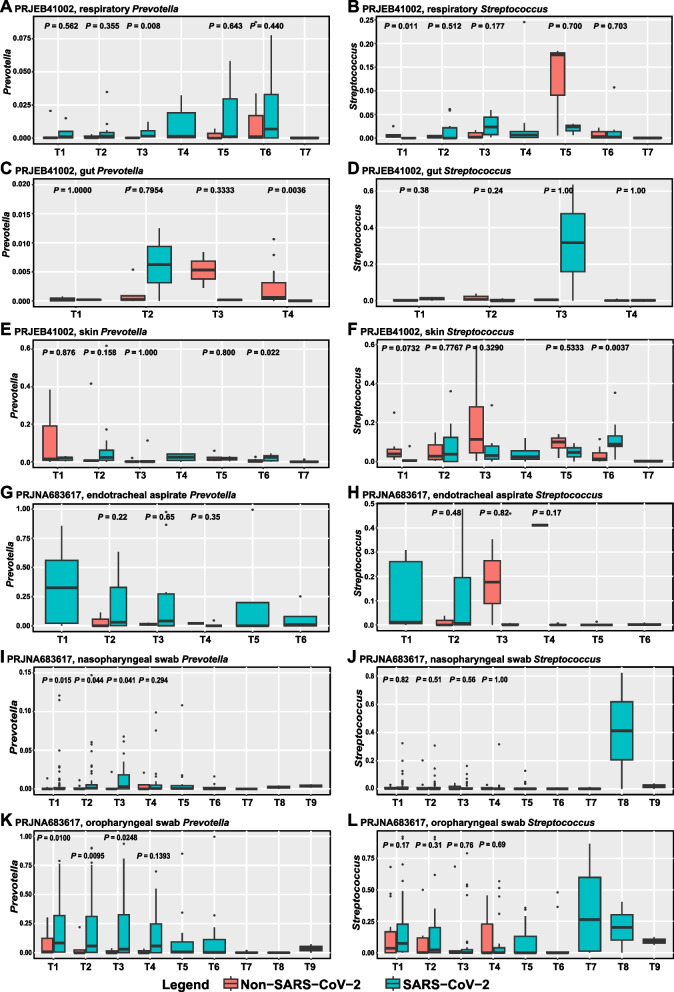
The boxplots showed the changes of *Prevotella* and *Streptococcus* in the gut, respiratory tract, and skin of SARS-CoV-2 and non-SARS-CoV-2 infected individuals at different observation time points (**A**-**L**)

**Fig. 5:**
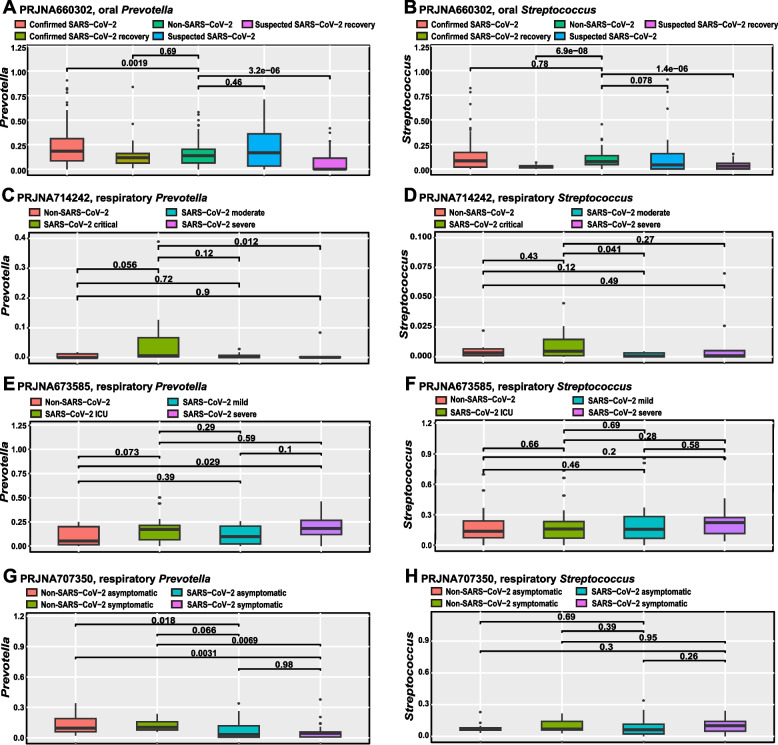
Boxplots of the changes of *Prevotella* and *Streptococcus* in SARS-CoV-2 infected individuals with different infection status (**A**-**H**)

At multiple time points in the longitudinal study of PRJEB41002, the abundance of *Prevotella* and *Streptococcus* in respiratory tract (Fig. 4A, B), gut (Fig. 4C, D), and skin (Fig. 4E, F) of SARS-CoV-2 infected individuals was higher than that of non-SARS-CoV-2 infected individuals. Similarly, in the respiratory longitudinal study PRJNA683617 (Fig. 4G-L), the trend of *Prevotella* and *Streptococcus* was still to enrich SARS-CoV-2 infected individuals. In addition, we found that *Prevotella* and *Streptococcus* were up-regulated in the oral cavity of confirmed SARS-CoV-2 infected individuals, but down-regulated in confirmed SARS-CoV-2 recovery individuals (Fig. 5A, B). In respiratory study PRJNA714242, *Prevotella* (*P* = 0.056) and *Streptococcus* (*P* = 0.43) were upregulated in critical SARS-CoV-2 infected individuals (Fig. 5C, D). In respiratory study PRJNA673585, *Prevotella* (*P* = 0.029) and *Streptococcus* (*P* = 0.2) were also enriched in severe SARS-CoV-2 infected individuals (Fig. 5E, F). However, in respiratory study PRJNA707350, *Prevotella* was significantly down-regulated in symptomatic (*P* < 0.01) and asymptomatic (*P* = 0.018) SARS-CoV-2 infected individuals (Fig. 5G, H).

## Discussion

Here, we reviewed the results of studies on the α-diversity of the human gut, respiratory, oral, and skin microbiota associated with SARS-CoV-2. We assembled the largest dataset available to date in order to assess the relationship between SARS-CoV-2 infection and the α-diversity of the human microbiota. Our meta-analysis revealed a significant down-regulation of the microbiota α-diversity in the gut and respiratory systems among individuals with SARS-CoV-2 infection, which is consistent with numerous current findings [8–12, 16, 29–34]. It should be noted that although state-of-the-art and widely used microbiological analysis software such as QIIME2 and the DADA2 algorithm were employed to mitigate heterogeneity in the processing and analysis of raw sequences from different studies, notable heterogeneity still existed among the included studies. For gut and respiratory studies, we observed that study type was one of the sources of heterogeneity. Cross-sectional and case–control studies typically involved samples collected at a single time point, while longitudinal studies consisted of samples collected at multiple time points. In gut studies, other sources of heterogeneity included antibiotics, country, and sequencing platform. In respiratory studies, sources of heterogeneity also encompassed sequencing regions. Previous studies have demonstrated that regional factors [35], antibiotic [36], gender [37], age [38], and diet [39] can influence the composition of the human microbiota. Due to limited access to open information included in this study, we were unable to analyze the sources of heterogeneity from additional perspectives. In summary, our study clarified that the α-diversity of gut and respiratory microbiota is downregulated after SARS-CoV-2 infection, providing readers with an understanding of the microbial characteristics of different human body sites after SARS-CoV-2 infection.

ML based on the human microbiota has been applied to predict various diseases and identify biomarkers. For example, it has been used to predict Vibrio cholerae infection [40], ulcerative colitis [41], and more. Similarly, the gut microbiota has shown promise in distinguishing the severity of COVID-19 [18] and effectively predicting protein markers for severe cases [42]. However, it remains to be explored whether the microbiota altered by SARS-CoV-2 infection can predict disease prognosis, including survival and death. In our study, we found that in the early stages of SARS-CoV-2 infection, alterations in the nasopharyngeal and oropharyngeal microbiota had the potential to predict patient survival and death. We observed that the predictive performance differed between the nasopharynx and oropharynx, as well as among different ML models. This suggests that when utilizing human microbiota to predict disease prognosis, we should consider the results from different body parts and ML models comprehensively. In the models we constructed, the AUC of the optimal model was only 0.847. This might be due to the small sample size and changes in microbiota characteristics following treatment for SARS-CoV-2 infection. Nonetheless, our study demonstrated the potential of ML based on human microbiota in predicting the prognosis of SARS-CoV-2-infected individuals, which may help in providing targeted treatment for severely SARS-CoV-2-infected individuals.

A study found that Dialister invisus ASV represents a unique case of overlap between the oral and gut microbiota in healthy individuals. Normally, the oral and gut microbiota differ under physiological conditions, and the presence of overlapping microbiota may indicate a certain pathological state [43]. In our study, we discovered genera such as *Prevotella* and *Streptococcus* that overlapped in the gut, respiratory tract, and oral cavity. Several studies have also demonstrated a significant up-regulation of *Prevotella * [44–47], *Streptococcus * [9, 44–46] and *Veillonella * [9, 44–46] following SARS-CoV-2 infection. *Prevotella*, a strictly anaerobic gram-negative bacillus, is known to be a major genus found in human skin, oral cavity, vagina, and gut [48]. It is frequently associated with respiratory tract infections, such as inhalation pneumonia [49] and pulmonary empyema [50]. Additionally, studies [51] have shown an increased abundance of *Prevotella* in the presence of viral infections associated with Human Immunodeficiency Virus, Papillomavirus, Herpesviridae, and respiratory viruses. Our study confirmed dysregulation of *Prevotella* in the human skin, oral cavity, gut, and respiratory tract after SARS-CoV-2 infection. Furthermore, *Prevotella* was found to be related to the severity and recovery of SARS-CoV-2 infection. Other studies have reported a correlation between long-lasting COVID-19 symptoms and elevated expression of oral *Prevotella * [52], which may be due to the ability of *Prevotella* to produce proteins that promote SARS-CoV-2 infection [53]. The precise mechanism by which *Prevotella* affects COVID-19 is not yet clear. However, previous research [54] has revealed that certain *Prevotella* strains can produce virulence factors that increase inflammatory response by activating Toll-like receptor 2 and inducing Th17-polarizing cytokines in antigen-presenting cells (such as IL-23 and IL-1), or stimulating epithelial cells to produce IL-8, IL-6, and CCL20. In summary, when individuals become ill due to the invasion of foreign pathogens, the normal human microbiota may be translocated and transformed into pathogenic bacteria, exacerbating the disease. The key bacteria *Prevotella* and *Streptococcus* proposed by us provide clues for future animal and in vitro experiments on SARS-CoV-2 infection intervention.

Our study has the following limitations: Firstly, due to the continuous updating of databases, our research may not reflect the latest research status. Secondly, the limited number of studies included in our analysis leads to certain limitations in generalizing our findings. Thirdly, there is a lack of animal and in vitro experimental validation for the key microbial communities we identified.

## Conclusions

Our study showed that SARS-CoV-2 infection was related to the down-regulation of α-diversity in the human gut and respiratory microbiota. The respiratory microbiota had the potential to predict the prognosis of individuals infected with SARS-CoV-2. *Prevotella* and *Streptococcus* might be key microbiota in SARS-CoV-2 infection.

### Supplementary Information

**Additional file 1:** **Supplementary Table 1. **Characteristics of included studies. 

**Additional file 2:** **Supplementary Figure 1.** Subgroup analysis of SARS-CoV-2 infection-associated gut microbiota studies. Forest plots for limiting factors were studies ruled out the effect of antibiotics (A), cross-sectional and case-control studies (B), Chinese studies (C), American studies (D), and Illumina MiSeq studies (E). **Supplementary Figure 2.** Subgroup analysis of SARS-CoV-2 infection-associated respiratory microbiota studies. Forest plots for limiting factors were cross-sectional and case-control studies (A), V4 sequencing studies (B). 

## Data Availability

The datasets used in the current study can be obtained from NCBI according to the Bioproject accession number mentioned in Supplementary Table 1.
