# Supplementary material for: Human microbiota dysbiosis after SARS-CoV-2 infection have the potential to predict disease prognosis
Source: BMC Infect Dis. 2023 Nov 29;23:841. doi: 10.1186/s12879-023-08784-x (PMC10685584; doi:10.1186/s12879-023-08784-x)
Supplement: Supplementary file 1 — Additional file 1: Supplementary Table 1. Characteristics of included studies. [file 12879_2023_8784_MOESM1_ESM.docx]

**Supplementary Table 1:** Characteristics of included studies.

|  | **Literature** | **Country** | **Sequencing Platforms / Regions** | **Available Bioproject for this paper** | **Study design** | **Results of α-diversity** | **Sample Type1** | **Sample Type2** |
| --- | --- | --- | --- | --- | --- | --- | --- | --- |
| 1 | Antonio Mazzarelli. et al 2021. 16S rRNA gene sequencing of rectal swab in patients affected by COVID-19. | Italy | Ion 530 chip by Ion S5 sequencer/ V2, V4, V8 and V3-6, 7–9 | Not available | Cross-sectional study Group1: 15 SARS-CoV-2 patients (9 ICU, 6 non-ICU) Group2: 8 non-SARS-CoV-2 patients (3 ICU, 5 non-ICU) | SARS-CoV-2 ICU VS SARS-CoV-2 non-ICU and non-SARS-CoV-2, Chao1 ↓ (significantly, s) | Rectal swab | Gut |
| 2 | Silan Gu. et al 2020. Alterations of the Gut Microbiota in Patients With Coronavirus Disease. et al 2019 or H1N1 Influenza. | China | Illumina MiSeq/V3-V4 | Not available | Cross-sectional study Group1: 30 patients with COVID-19 Group2: 24 patients with influenza A(H1N1) Group3: 30 healthy controls (HCs) | Group1 and Group2 VS Group3, Shannon ↓ (s) and Chao ↓ (s) | Fecal samples |  |
| 3 | Wanyin Tao. et al 2020. Analysis of the intestinal microbiota in COVID-19 patients and its correlation with the inflammatory factor IL-18. | China | Illumina MiSeq/V4 | Not available | Cross-sectional study Group1: 62 COVID-19 patients Group2: 33 seasonal flu patients  Group3: 40 healthy controls | Group1 VS Group2 and Group3, Chao1 ↓ (s) | Fecal samples |  |
| 4 | Gergely Babszky. et al 2021. COVID-19 Infection Alters the Microbiome: Elite Athletes and Sedentar1 Patients Have Similar Bacterial Flora. | Hungary | Ion PGM Hi-Q view OT2 Kit | Not available | Longitudinal study Group1: 20 active competing athletes (CA) with COVID-19 Group2: 20 sedentary subjects (SED) with COVID-19 | The Shannon diversity of the microbiome was independent from training status or COVID-19 infection. | Fecal samples |  |
| 5 | Mahejibin Khan. et al 2021. Gut Dysbiosis and IL-21 Response in Patients with Severe COVID-19. | India | Illumina Genome Analyzer II/V3-V4 | PRJNA705797 | Cross sectional prospective study Group1: 30 COVID-19 patients (Asymptomatic, Mild, Severe) Group2: 10 healthy controls | Severity of symptoms ↑ , ACE ↓ , InvSimpson ↓ and Fisher ↓ (Significance not described, snd) | Fecal samples |  |
| 6 | André Moreira-Rosário. et al 2021. Gut Microbiota Diversity and C-Reactive Protein Are Predictors of Disease Severity in COVID-19 Patients. | Portugal | Ion S5 System/V3 and V4 | Not available | Cross sectional prospective study Group1: 19 mild COVID-19 patients Group2: 37 moderate COVID-19 patients  Group3: 59 severe COVID-19 patients | WHO score ↑ , Shannon ↓ (not significantly, ns)；COVID-19 ICU patients VS ward/hospitalized COVID-19 patients, Shannon ↓ (snd); The feces of SARS-CoV-2 positive patients VS negative, Shannon ↓ (Not significantly, ns). | Fecal samples |  |
| 7 | Yu Tian. et al 2021. Gut Microbiota May Not Be Fully Restored in Recovered COVID-19 Patients After 3-Month Recovery. | China | Illumina MiSeq/V3–V4 | PRJNA683685 | Cross-sectional study Group1: 7 recovered COVID-19 patients Group2: 7 healthy controls | Group1 VS Group2, Chao1 ↓ (s)，Simpson ↑ (s), other ns | Fecal samples |  |
| 8 | Longxian Lv. et al 2021. Gut mycobiota alterations in patients with COVID-19 and H1N1 infections and their associations with clinical features. | China | Illumina MiSeq/not described | PRJNA636824 | Cross-sectional study, gut mycobiota  Group1: 67 COVID-19 patients Group2: 35 H1N1-infected patients  Group3: 48 healthy controls | Group1 VS Group3, Chao1 ↓ (s)，Shannon ns | Fecal samples |  |
| 9 | Graham J. Britton. et al 2021. Limited intestinal inflammation despite diarrhea, fecal viral RNA and SARS-CoV-2-specific IgA in patients with acute COVID-19. | USA | Illumina HiSeq/V2 | Not available | Longitudinal study 44 symptomatic COVID-19 patients | (1) Shannon in mild, moderate or severe COVID-19 patients ns. (2) Shannon in COVID-19 patients with and without diarrhea and as relates to the Bristol score of each sample. (3) Shannon ↓ (s) in hospitalized COVID-19 patients recently or currently receiving antibiotic therapy. | Fecal samples |  |
| 10 | Yaya Zhou. et al 2021. Linking the gut microbiota to persistent symptoms in survivors of COVID-19 after discharge. | China | Illumina MiSeq/V3–V4 | PRJNA736160 | Cross-sectional study Group1: 15 recovered healthcare workers (HCWs) with COVID-19 Group2: 14 healthy controls (HCs) | Group1 VS Group2, Shannon ↓, Sobs ↓ (ns), Chao ↓ (ns), Ace ↓ (ns). | Fecal samples |  |
| 11 | Han-Na Kim. et al 2021. Reversion of Gut Microbiota during the Recovery Phase in Patients with Asymptomatic or Mild COVID-19: Longitudinal Study. | Korea | Illumina MiSeq/V3 and V4 | Not available | Longitudinal study Group1: 12 COVID-19 patients (infected state (respiratory positive, RP), recovered state (respiratory negative, RN)) Group2: 36 healthy controls | RP VS RN, Observed ↓ (ns) and Faith’s phylogenetic diversity ↓ (ns), Shannon ↓ (ns), and Pielou’s evenness ↓ (s). | Fecal samples |  |
| 12 | Yanfei Chen. et al 2021. Six-month follow-up of gut microbiota richness in patients with COVID-19. | China | Illumina MiSeq/V3-4 | Not available | Longitudinal study Group1: 30 COVID-19 patients (acute, convalescence, postconvalescence) Group2: 30 non-COVID-19 controls | (1) Group1 (acute, convalescence, postconvalescence) VS Group2, Chao1 ↓ (s). (2) There was a non-significant increase of the Chao1 index from the acute phase to the convalescence and postconvalescence. | Fecal samples |  |
| 13 | Longxian Lv. et al 2021. The faecal metabolome in COVID-19 patients is altered and associated with clinical features and gut microbes | China | Illumina MiSeq/not described | PRJNA636824 | Cross-sectional study Group1: 56 COVID-19 patients Group2: 47 healthy controls | Not described. | Fecal samples |  |
| 14 | Rachel C Newsome. et al 2021. The gut microbiome of COVID-19 recovered patients returns to uninfected status in a minority-dominated United States cohort. | USA | Illumina MiSeq/V1-V3 | PRJNA678695 | Cross-sectional study Group1: 50 SARS-CoV-2 infected patients Group2: 9 SARS-CoV-2 recovered patients Group3: 34 non-SARS-CoV-2 subjects | Shannon (ns), Group1 > Group2 > Group3 | Fecal samples |  |
| 15 | Paolo Gaibani. et al 2021. The Gut Microbiota of Critically Ill Patients With COVID-19. | Italy | Illumina MiSeq/V3-V4 | Not available | Cross-sectional study Group1: 69 COVID-19 patients (ICU and bloodstream infection (BSI)) Group2: 69 healthy controls Group3: 16 non-COVID-19 ICU patients | (1) Group1 VS Group2, Simpson ↓ (s). (2) ICU and BSI COVID-19 patients VS non-ICU and non-BSI COVID-19 patients, Observed ↓ (s). | Fecal samples |  |
| 16 | Zhao Zhang. et al 2021. The Potential Role of an Aberrant Mucosal Immune Response to SARS-CoV-2 in the Pathogenesis of IgA Nephropathy. | China | Illumina MiSeq/V3 | Not available | Longitudinal study Group1: 88 COVID-19 patients Group2: 5 healthy controls | Intestinal dysbiosis and inflammation were observed in the COVID-19 IgAN case. | Fecal samples |  |
| 17 | 2021, no articles found | Morocco | Illumina MiSeq/V3-V4 | PRJNA728736 | Group1: 4 COVID-19 patients Group2: 4 non-COVID-19 patients | Not described. | Fecal samples |  |
| 18 | Zhigang Ren. et al 2021. Acute SARS-CoV-2 infection is associated with an increased abundance of bacterial pathogens, including Pseudomonas aeruginosa in the nose. | USA | Illumina MiSeq/V4 | Not available | Cross-sectional study Group1: 21 Non-SARS-CoV-2 patients  Group2: 45 Non-SARS-CoV-2 healthcare workers (HCW)  Group3: 68 SARS-CoV-2 patients | Group3 VS Group2, Observed ↓ (s)；Group3 VS Group1, Observed ↑ (s). | Nose swab | Respiratory tract |
| 19 | Jillian H Hurst. et al 2021. Age-related changes in the upper respiratory microbiome are associated with SARS-CoV-2 susceptibility and illness severity. | USA | Illumina MiSeq/V4 | Not available | Prospective cohort study Group1: 75 Non-SARS-CoV-2 Group2: 88 SARS-CoV-2 no respiratory symptom Group3: 111 SARS-CoV-2 with respiratory symptoms | (1) Non-SARS-CoV-2 VS SARS-CoV-2, Shannon ns. (2) SARS-CoV-2 VS Non-SARS-CoV-2, Chao1 ↑ (s). | Nose swab |  |
| 20 | Ravindra Kolhe. et al 2021. Alteration in Nasopharyngeal Microbiota Profile in Aged Patients with COVID-19. | USA | Illumina MiSeq/V4-V5 | Not available | Cross-sectional study Group1: 27 Non-SARS-CoV-2 no symptoms Group2: 30 SARS-CoV-2 no symptoms Group3: 27 SARS-CoV-2 with symptoms | (1) Group2 VS Group1, Shannon ↑ (s), Observed ↑ (s), Chao1 ↑ (s). (2) Group3 VS Group1, Observed ↑ (s), Chao1 ↑ (s). | Nose swab |  |
| 21 | Martina Rueca. et al 2021. Investigation of Nasal/Oropharyngeal Microbial Community of COVID-19 Patients by 16S rDNA Sequencing. | Italy | Ion S5 Sequencer/V2-4-8 and V3-6 and 7-9 | Not available | Cross-sectional study Group1: 10 SARS-CoV-2 ICU Group2: 11 SARS-CoV-2 Pauci Group3: 10 negative Controls Group4: 8 Other HCoVs | Group1 VS Group2 and Group3, Chao1 ↓ (s), Shannon ↓ (s). | Nasal/oropharyngeal swabs |  |
| 22 | Maria Paz Ventero. et al 2021. Nasopharyngeal Microbial Communities of Patients Infected With SARS-CoV-2 That Developed COVID-19. | Spain | Illumina MiSeq/V3-V4 | PRJNA673585 | Cross-sectional study Group1: 18 SARS-CoV-2 negative patients  Group2: 19 COVID-19 patients with mild symptoms but no later hospital admission Group3: 18 COVID-19 patients severe symptoms followed by hospital admission Group4: 19 COVID-19 patients with severe symptoms which were eventually admitted into intensive care units (ICU) | The richness and diversity indexes ns among groups. | Nasopharyngeal swabs |  |
| 23 | Abhishek Gupta. et al 2022. Nasopharyngeal microbiome reveals the prevalence of opportunistic pathogens in SARS-CoV-2 infected individuals and their association with host types. | India | Illumina MiSeq/V4 | PRJNA707350 | Cross-sectional study Group1: 63 SARS-CoV-2 infected Group2: 26 non-infected individuals | Group1 VS Group2, Simpson and Shannon ns；Observed OTUs and Chao1 ↓ (s). | Nasopharyngeal swabs |  |
| 24 | Phillip A Engen. et al 2021. Nasopharyngeal Microbiota in SARS-CoV-2 Positive and Negative Patients. | USA | Illumina MiSeq/V4 | PRJNA704967 | Cross-sectional study Group1: 9 COVID-19-positive patients Group2: 10 COVID-19-negative patients | The Shannon, Simpson, Observed, Evenness indexes ns between groups. | Nasopharyngeal swabs |  |
| 25 | Flavio De Maio. et al 2021. Nasopharyngeal Microbiota Profiling of SARS-CoV-2 Infected Patients. | Italy | Illumina MiSeq/V5–V6 | Not available | Cross-sectional study Group1: 18人SARS-CoV-2 positive patients Group2: 22 SARS-CoV-2 negative patients | Group1 VS Group2, Observed ↑ (ns), Shannon ↑ (ns), and Inverse-Simpson ↑ (ns). | Nasopharyngeal swabs |  |
| 26 | Tzipi Braun. et al 2021. SARS-CoV-2 does not have a strong effect on the nasopharyngeal microbial composition. | Israel | Illumina MiSeq/V4 | Not available | Longitudinal study 33 confirmed or suspect for COVID-19 subjects 29 SARS-CoV-2 negative samples and 26 SARS-CoV-2 positive samples | SARS-CoV-2 positive samples VS negative samples, Faith's phylogenetic diversity ↑ (ns), Shannon ↑ (ns) and evenness ↑ (ns). | Nasopharyngeal swabs |  |
| 27 | Christian Rosas-Salazar. et al 2021. SARS-CoV-2 infection and viral load are associated with the upper respiratory tract microbiome. | USA | Illumina MiSeq/V4 | Not available | Cross-sectional study Group1: 38 SARS-CoV-2 infected Group2: 21 non-infected individuals | Group1 VS Group2, Observed species ↑ (s), shannon ↑ (ns), Inverse Simpson ↑ (ns). | Nasopharyngeal swabs |  |
| 28 | Carter Merenstein. et al 2021. Signatures of COVID-19 severity and immune response in the respiratory tract microbiome. | USA | ETA: HiSeq 2500/V1, V2; OP, NP: Roche 454 GS-FLX/V1, V2 | PRJNA683617 | Longitudinal study Group1: 83 hospitalized COVID-19 patients Group2: 13 Non-COVID critically ill patients Group3: 30 healthy controls | (1) In oropharyngeal samples of COVID-19 patients, WHO score ↑ , Simpson ↓ (s). (2) In endotracheal aspirate samples, Group1 VS Group3, Simpson ↓ (s). | Oropharyngeal (OP) and nasopharyngeal (NP) swabs, and endotracheal aspirates (ETA) |  |
| 29 | Alejandra Hernández‑Terán. et al 2021. Dysbiosis and structural disruption of the respiratory microbiota in COVID-19 patients with severe and fatal outcomes | Mexico | Illumina MiSeq/V3–V4 | PRJNA726205 | Cross-sectional study Group1: 37 mild COVID-19 Group2: 27 severe COVID-19 Group3: 19 fatal COVID-19 Group4: 7 healthy controls Group5: 5 non-COVID-19-pneumonia | Group3 VS Group4 and Group2, Shannon-Wiener index ↓ (s). | Upper respiratory samples, initial respiratory samples (oropharyngeal swabs, nasopharyngeal swabs,  and tracheal aspirates) |  |
| 30 | Nikaïa Smith. et al 2021. Distinct systemic and mucosal immune responses during acute SARS-CoV-2 infection | France | Illumina MiSeq/V3–V4 | PRJNA714242 | Non-interventional study Group1: 10 healthy controls COVID-19 Group2: 10 mild-to-moderate COVID-19 Group3: 10 severe COVID-19 Group4: 12 critical COVID-19 | Simpson and Shannon diversity indexes, severe and critical COVID-19 ↓ (snd). | Nasopharynx swabs |  |
| 31 | 2020, no articles found | USA | Illumina MiSeq/V4 | PRJEB41721 | Group1: 15 SARS-CoV-2 positive samples Group2: 6 SARS-CoV-2 negative samples | Not described. | nasopharyngeal |  |
| 32 | 2021, no articles found | USA | llumina MiSeq/V4 | PRJNA726992 | Group1: 38 COVID-19 samples Group2: 28 non-COVID-19 samples | Not described. | Upper respiratory |  |
| 33 | Emily Happy Miller. et al 2021. Oral Microbiome Alterations and SARS-CoV-2 Saliva Viral Load in Patients with COVID-19. | USA | Illumina MiSeq/V1-V2 | PRJNA669421 | Cross-sectional study Group1: 53 COVID-19 patients Group2: 59 non-COVID-19 patients | Group1 VS Group2, Shannon ↑ (ns), Chao1 ns. | Saliva | Oral cavity |
| 34 | Ram Prasad. et al 2021. Plasma microbiome in COVID-19 subjects: an indicator of gut barrier defects and dysbiosis. | USA | Illumina MiSeq/V3-V4 | Not available | Cross-sectional study Group1: 30 COVID-19 patients  Group2: 16 healthy control | Group1 VS Group2, Pielou’s evenness, Faith’s phylogenetic diversity, Observed OTUs, and Shannon’s Dysbiosis indexes ns. | Plasma sample | Plasma |
| 35 | Zhigang Ren. et al 2021. Alterations in the human oral and gut microbiomes and lipidomics in COVID-19. | China | Illumina MiSeq/V3-V5 | PRJNA660302 | Prospective specimen collection and retrospective blinded evaluation design Group1: 146 confirmed COVID-19 patients (CPs) Group2: 37 suspected COVID-19 patients (SPs) with IgG positivity  Group3: 22 CPs who recovered (CPRs) Group4: 37 paired SPs who recovered (SPRs) Group5: 150 Healthy controls (HCs) | CPs VS HCs, CPs oral Shannon ↓ (s) and Simpson ↓ (s), gut Shannon ↓ (s) | Tongue-coating and faecal samples | Multiple types of sample |
| 36 | Yongjian Wu. et al 2021. Altered oral and gut microbiota and its association with SARS-CoV-2 viral load in COVID-19 patients during hospitalization. | China | Illumina NovaSeq 6000/V3–V4 | PRJNA684070 | Longitudinal study Group1: 53 COVID-19 patients Group2: 76 healthy controls | Group1 VS Group2, respiratory tract and gut Faith's phylogenetic diversity ↓ (s). | Throat swab and fecal samples |  |
| 37 | Clarisse Marotz. et al 2020. Microbial context predicts SARS-CoV-2 prevalence in patients and the hospital built environment. | USA | Illumina MiSeq/V4 | PRJEB41002 | Longitudinal study Group1: 16 SARS-CoV-2 infected patients(118 samples) Group2: 10 health care workers(113 samples) Group3: 734 hospital surfaces samples | (1) Faith’s phylogenetic diversity ↑ (s) among surface samples than patient or health care worker samples. (2) Faith’s phylogenetic diversity ↑ (s) in SARS-CoV-2 positive samples of forehead, inside floor, and outside floor samples. | Forehead, nares, stool samples; throat swabs and/or tracheal aspirate samples; oral samples; floor, door handle, bedrail, keyboard, air filter intake, patient care equipment. |  |
| 38 | Valerio Iebba. et al 2021. Profiling of Oral Microbiota and Cytokines in COVID-19 Patients. | Italy | Ion Torrent PGM/V2–V3 | PRJNA692359 | Cross-sectional study Group1: 26 naive severe COVID-19 patients Group2: 15 Healthy controls | Group1 VS Group2, oral microbiota species richness ↓ (s)，Shannon ns. | oral swab specimens touching the tongue, palatum |  |
| 39 | Rong Xu. et al 2021. Progressive deterioration of the upper respiratory tract and the gut microbiomes in children during the early infection stages of COVID-19. | China | Illumina-P250 sequencer/V4 | Not available | Longitudinal study Group1: 9 COVID-19 children patients Group2: 14 healthy control children | Group1 VS Group2, the throat and nasal swabs had significantly lower richness; the gut microbiome had significantly higher evenness. | Nasal swabs, throat swabs, and feces |  |
| 40 | Rong Xu. et al 2021. Temporal association between human upper respiratory and gut bacterial microbiomes during the course of COVID-19 in adults. | China | Illumina MiSeq/V4 | PRJNA639286 | Longitudinal study Group1: 35 COVID-19 patients (community types I-IV) Group2: 19 healthy adult controls (community type H) Group3: 10 non-COVID-19 patients (community type NP) | (1) In throat swabs sample: Group1(II-IV) VS Group2, observed ↓ (s); Group1(III-IV) VS Group2, observed ↓ (s), Pielou’s evenness ↓ (s); Group1(I-II) VS Group2, observed ↑ (s), Pielou’s evenness ↑ (s); (2) Gut microbiome from the lower-diversity community type (II or III) towards a higher-diversity type (I or II). | Throat and anal swabs |  |

Footnotes: ↓, down-regulation; ↑, up-regulation.
