# Supplementary material for: Human microbiota dysbiosis after SARS-CoV-2 infection have the potential to predict disease prognosis
Source: BMC Infect Dis. 2023 Nov 29;23:841. doi: 10.1186/s12879-023-08784-x (PMC10685584; doi:10.1186/s12879-023-08784-x)
Supplement: Supplementary file 2 — Additional file 2: Supplementary Figure 1. Subgroup analysis of SARS-CoV-2 infection-associated gut microbiota studies. Forest plots for limiting factors were studies ruled out the effect of antibiotics (A), cross-sectional and case-control studies (B), Chinese studies (C), American studies (D), and Illumina MiSeq studies (E). Supplementary Figure 2. Subgroup analysis of SARS-CoV-2 infection-associated respiratory microbiota studies. Forest plots for limiting factors were cross-sectional and case-control studies (A), V4 sequencing studies (B). [file 12879_2023_8784_MOESM2_ESM.docx]

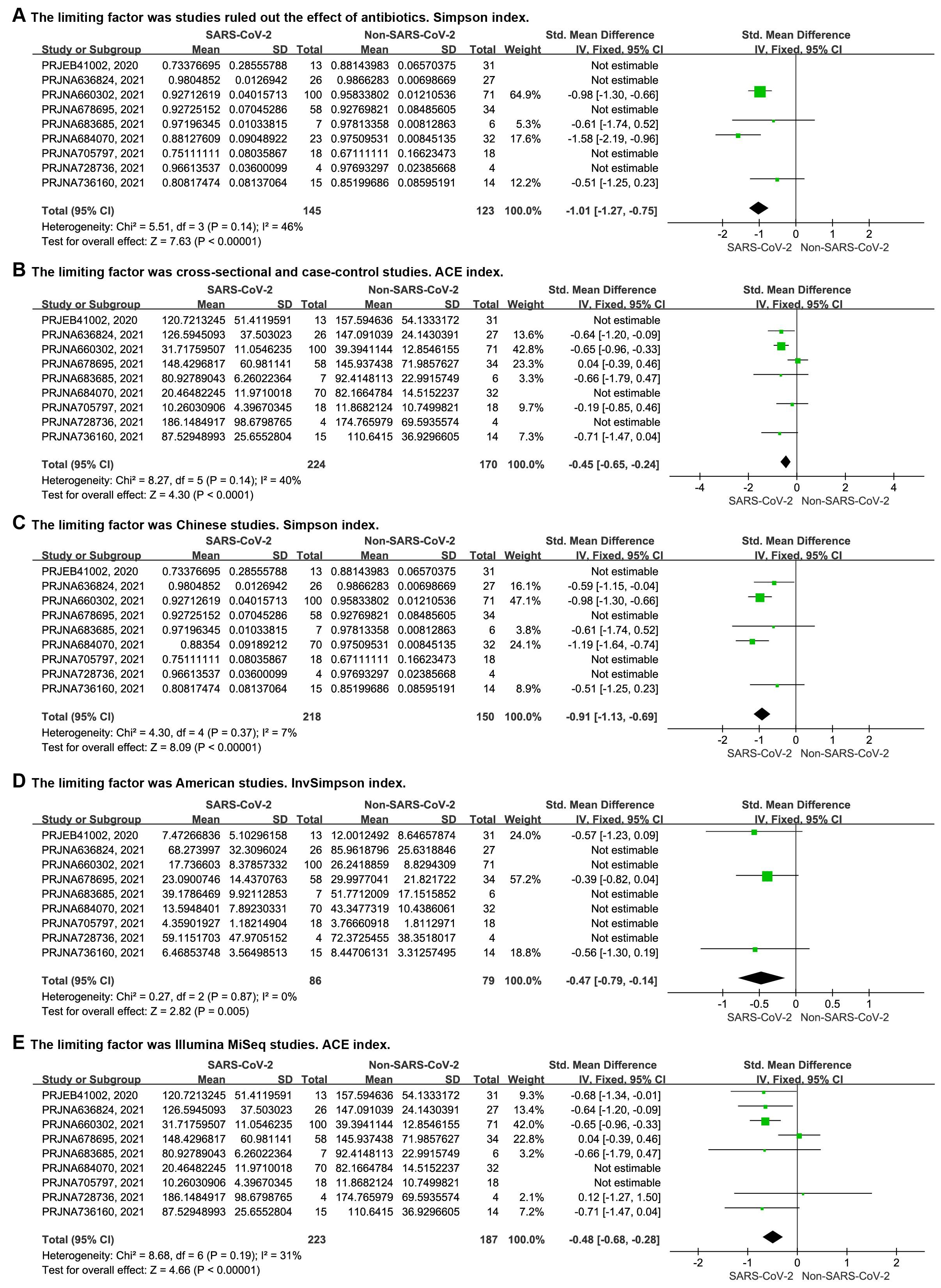


**Supplementary Figure 1.** Subgroup analysis of SARS-CoV-2 infection-associated gut microbiota studies. Forest plots for limiting factors were studies ruled out the effect of antibiotics (A), cross-sectional and case-control studies (B), Chinese studies (C), American studies (D), and Illumina MiSeq studies (E).


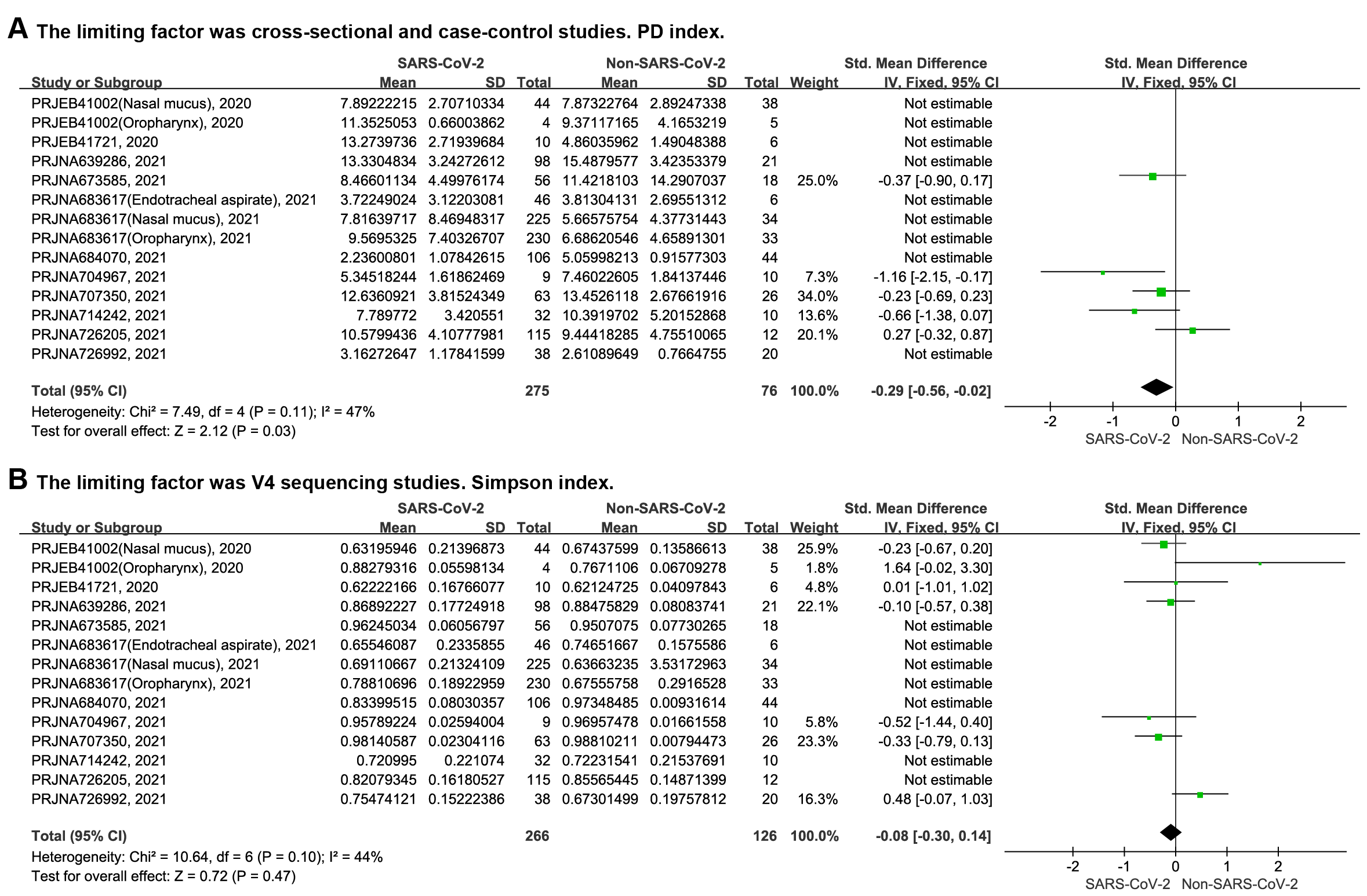


**Supplementary Figure 2.** Subgroup analysis of SARS-CoV-2 infection-associated respiratory microbiota studies. Forest plots for limiting factors were cross-sectional and case-control studies (A), V4 sequencing studies (B).
